# Supplementary material for: Malaria surveillance reveals parasite relatedness, signatures of selection, and correlates of transmission across Senegal
Source: Nat Commun. 2023 Nov 10;14:7268. doi: 10.1038/s41467-023-43087-4 (PMC10638404; doi:10.1038/s41467-023-43087-4)
Supplement: Supplementary file 1 — Supplementary Information [file 41467_2023_43087_MOESM1_ESM.pdf]

Supplementary Figures

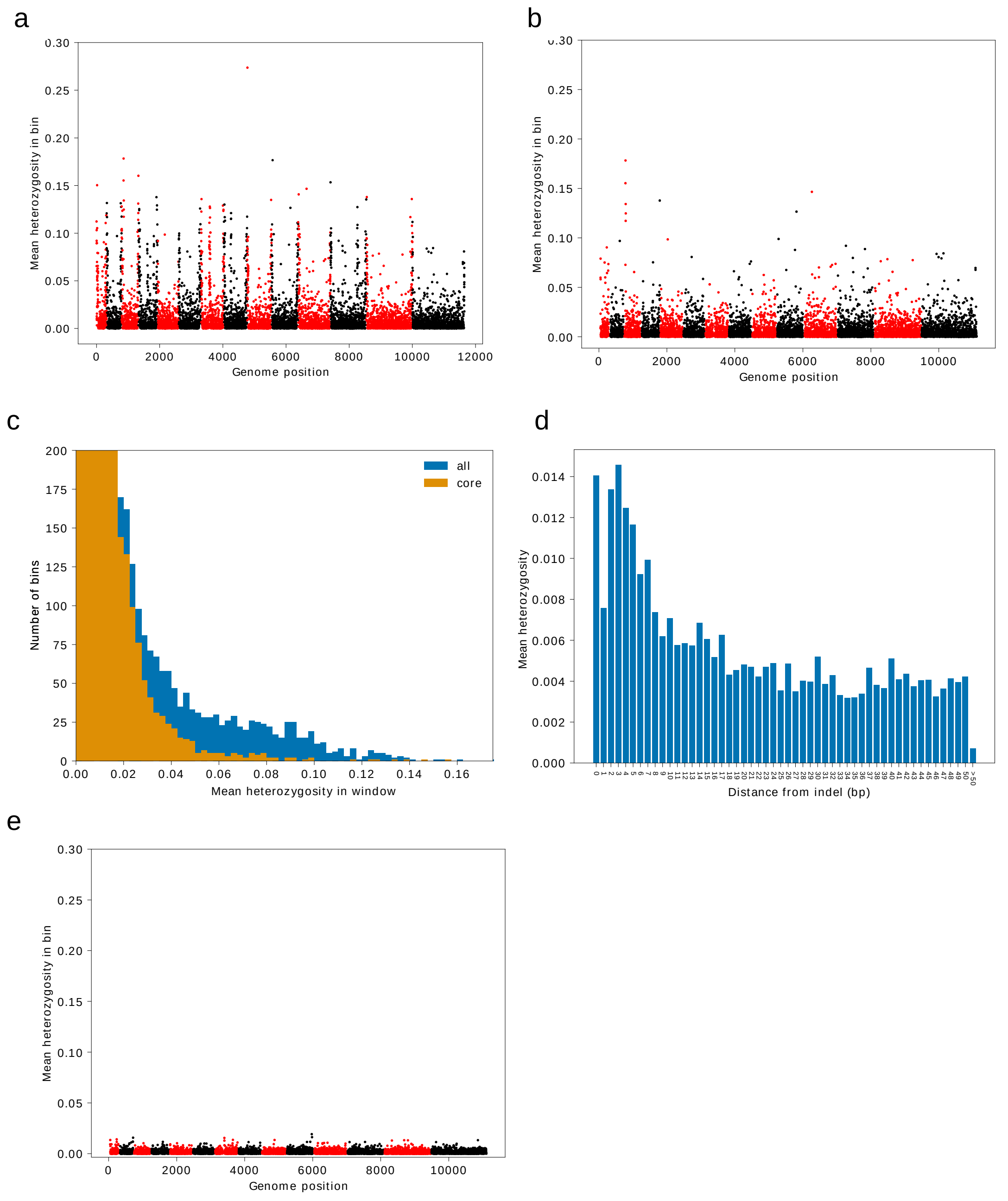

Supplementary Figure 1. Steps in selecting SNP sites for analysis, based on the complete Pf3K dataset. a) Mean rate of heterozygous calls for nonoverlapping 2 kb windows across the P. falciparum *genome. b) Same as (a) but restricted to 'core' regions of the genome [1]. c) Distribution of bin heterozygosities with and without non-core regions. d) Heterozygosity as a function of distance from known indels; '0' bin contains sites within indels. e) Rate of heterozygous calls after application of all filters.*

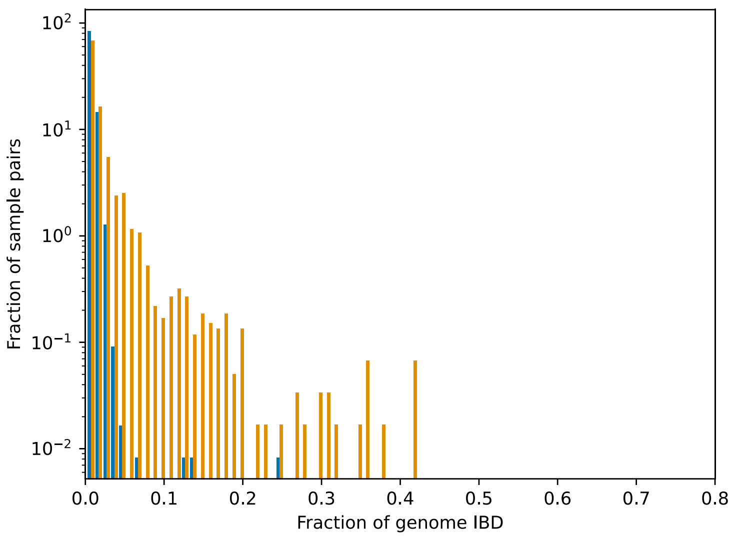

Supplementary Figure 2. IBD fraction for sample pairs enriched (orange) and depleted (blue) for relatedness; only higher coverage samples shown.

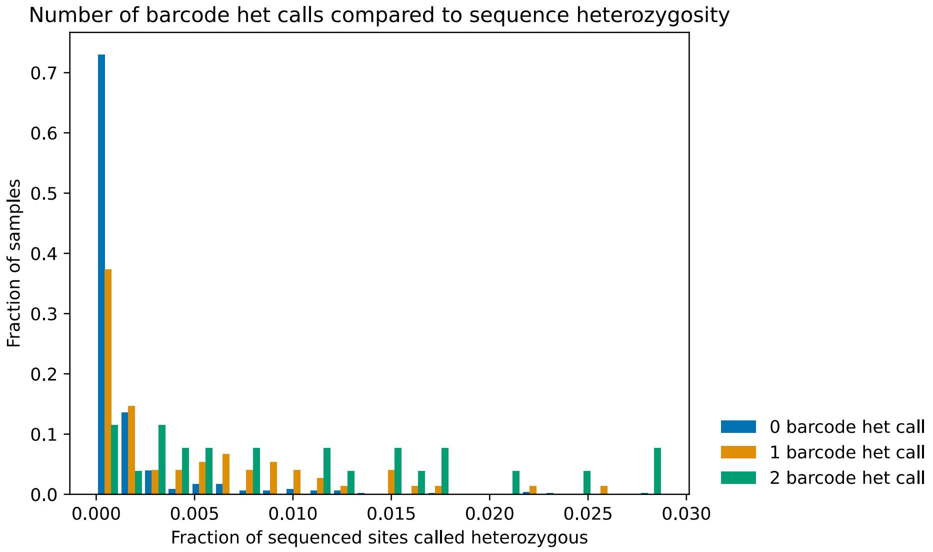

Supplementary Figure 3. Comparison of complexity of infection measures from barcode and sequence data. Shown is the distribution of sequenced sites that were called as heterozygous for samples with both barcode and whole sequence data. Samples with 0 or 1 heterozygous barcode sites were classified as probable monogenomic.

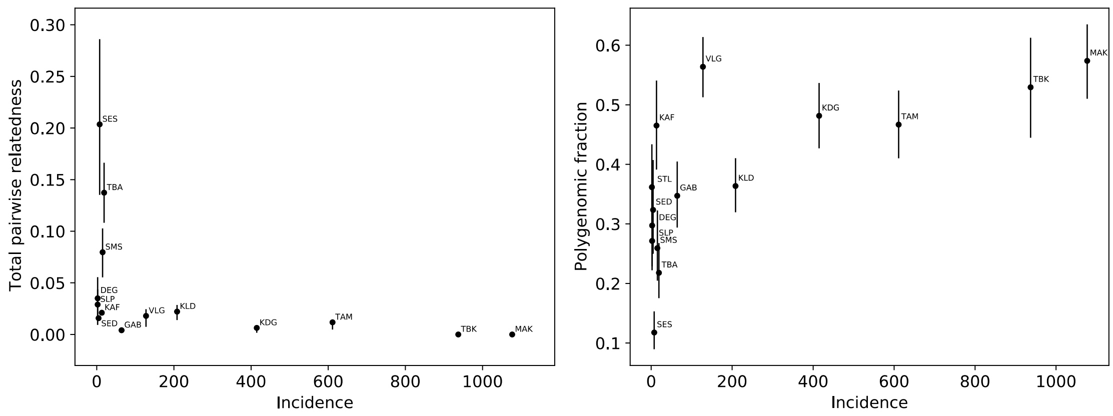

Supplementary Figure 4. Total pairwise relatedness and polygenomic fraction vs reported incidence (per 1000 per year) at study site (linear scale). See Table 1 for site codes and sample sizes; error bars represent 68% confidence intervals, see Methods.

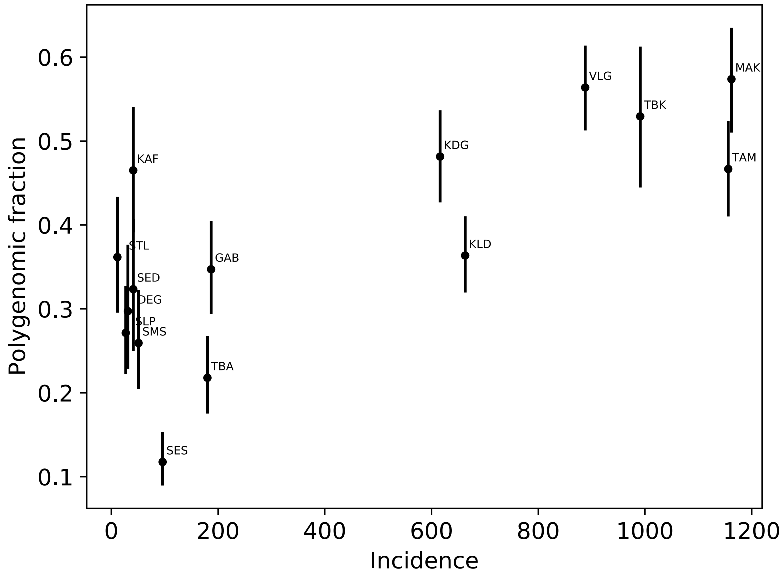

Supplementary Figure 5. Polygenomic fraction as a function of estimated incidence (per 1000 per year), where the incidence has been adjusted for care seeking, testing of suspected cases, and reporting completeness. See Table 1 for site codes and sample sizes; error bars represent 68% confidence intervals, see Methods.

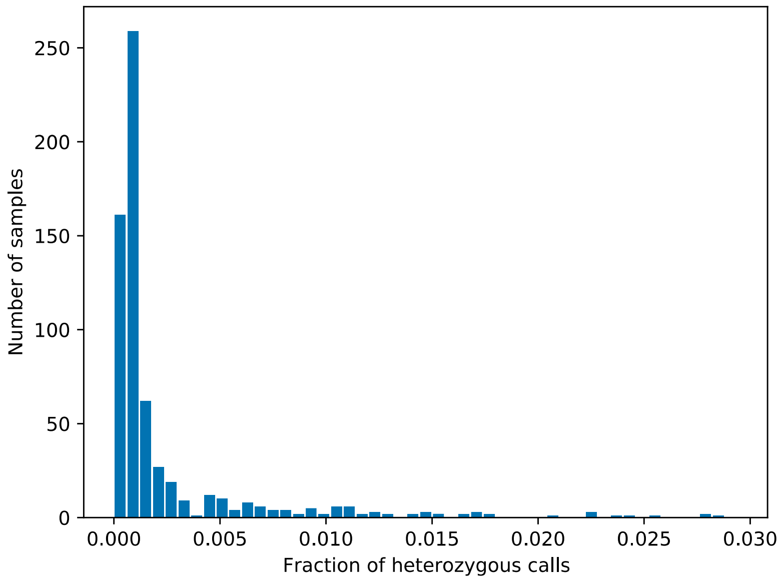

Supplementary Figure 6. Fraction of heterozygous genotypes (out of all called sites) for all sequenced samples. Samples with a fraction > 0.0024 were considered possibly polygenomic and excluded from further analysis.

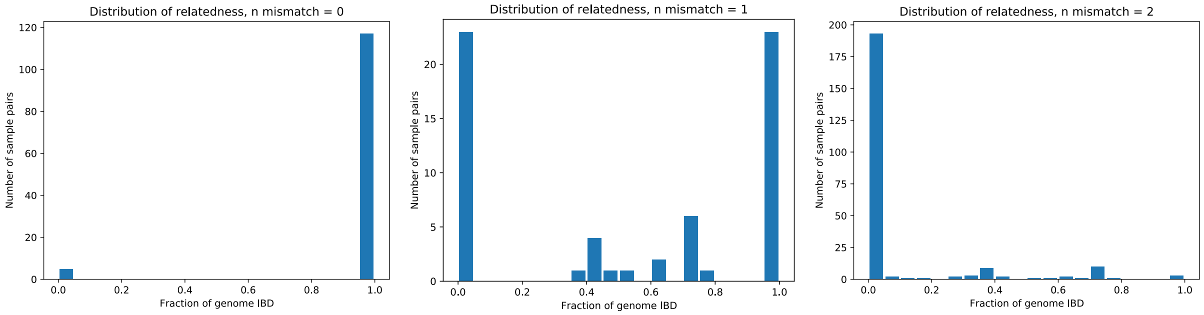

Supplementary Figure 7. Accuracy of relatedness inference from barcode data. The panels show the distribution of genome wide IBD fraction as determined from sequence data for sample pairs with 0, 1, and 2 barcode mismatches, respectively,

Genetic measures in model	R^2	Adjusted R^2
polygenomic fraction (poly)	0.564	0.527
total relatedness (total)	0.194	0.127
poly + total	0.619	0.550
poly + clonal	0.590	0.515
poly + partial	0.593	0.519
poly + clonal + partial	0.621	0.507

Supplementary Table 1. Performance of different genetic measures as predictors of malaria incidence at each site, as determined by regressing log(incidence) on the indicated measures. Clonal: clonal relatedness; partial: partial relatedness.

Supplementary Information: IBD pileup plots for all chromosomes

The fraction of sample pairs that are IBD as a function of genomic position, split into samples that are (“within cluster”) or are not (“outside cluster”) part of the large network of related parasites. Individual chromosomes are displayed (chromosome 1 – 14) with the position of some individual genes identified by green dotted line including: dhfr (Pfdhfr, chromosome 4), mdr1 (Pfmdr1, chromosome 5), aat1 (Pfaat1, chromosome 6), crt (Pfcrt, chromosome 7), dhps (Pfdhps, chromosome 8), gch1 (Pfgch1, chromosome 12), and fd (ferredoxin, chromosome 13).

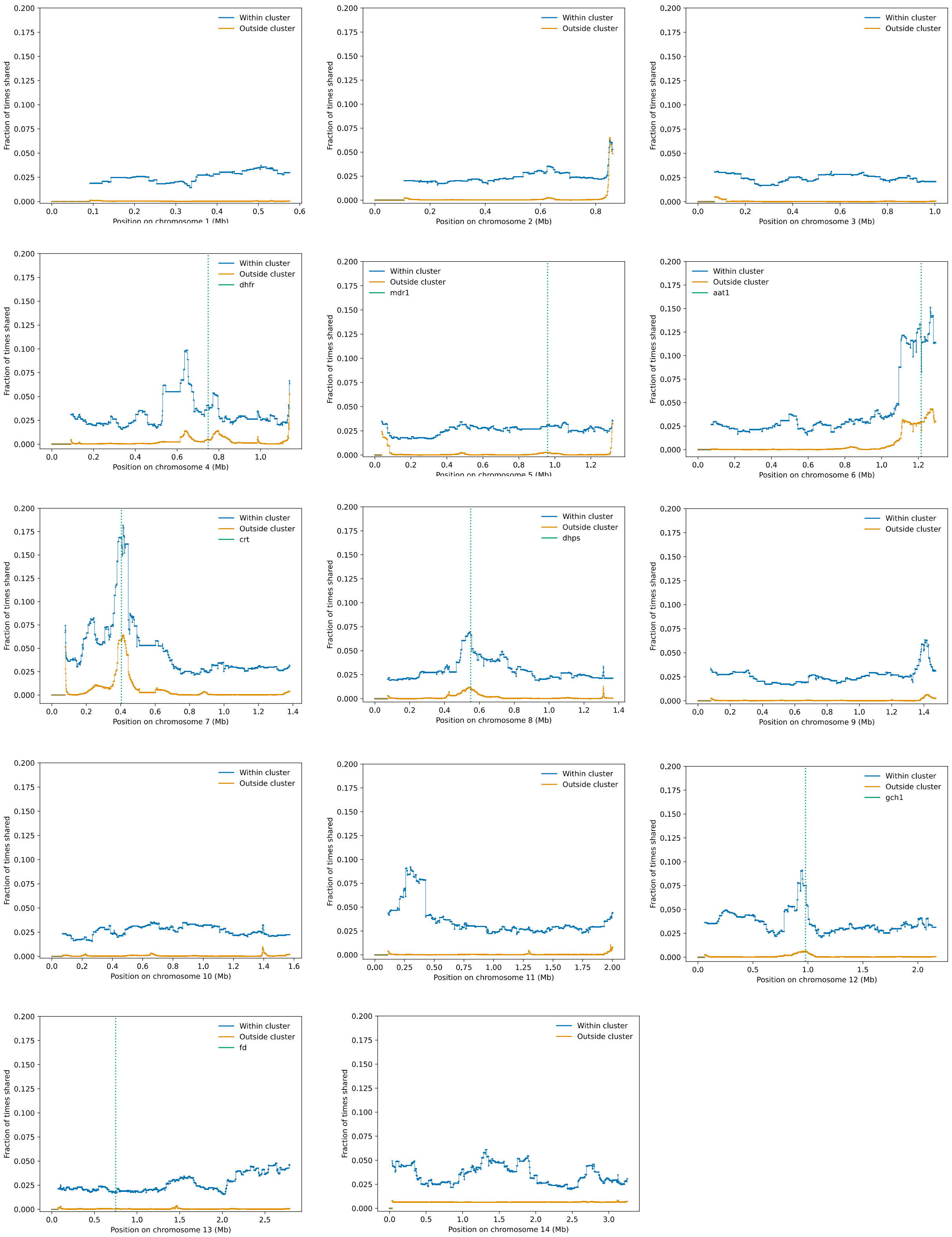

Fraction of times shared
Within cluster
Outside cluster
Position on chromosome 1 (Mb)
Position on chromosome 2 (Mb)
Position on chromosome 3 (Mb)
dhfr
Position on chromosome 4 (Mb)
mdr1
Position on chromosome 5 (Mb)
aat1
Position on chromosome 6 (Mb)
crt
Position on chromosome 7 (Mb)
dhps
Position on chromosome 8 (Mb)
Position on chromosome 9 (Mb)
Position on chromosome 10 (Mb)
Position on chromosome 11 (Mb)
gch1
Position on chromosome 12 (Mb)
fd
Position on chromosome 13 (Mb)
Position on chromosome 14 (Mb)
