# Supplementary material for: Malaria surveillance reveals parasite relatedness, signatures of selection, and correlates of transmission across Senegal
Source: Nat Commun. 2023 Nov 10;14:7268. doi: 10.1038/s41467-023-43087-4 (PMC10638404; doi:10.1038/s41467-023-43087-4)
Supplement: Supplementary file 3 — Reporting Summary [file 41467_2023_43087_MOESM3_ESM.pdf]

nature portfolio

Corresponding author(s): Sarah K. Volkman, ScD FASTMH

Last updated by author(s): Oct 16, 2023

Reporting Summary

Nature Portfolio wishes to improve the reproducibility of the work that we publish. This form provides structure for consistency and transparency in reporting. For further information on Nature Portfolio policies, see our Editorial Policies and the Editorial Policy Checklist.

Statistics

For all statistical analyses, confirm that the following items are present in the figure legend, table legend, main text, or Methods section.

n/a	Confirmed	
☐	☒	The exact sample size (*n*) for each experimental group/condition, given as a discrete number and unit of measurement
☐	☒	A statement on whether measurements were taken from distinct samples or whether the same sample was measured repeatedly
☐	☒	The statistical test(s) used AND whether they are one- or two-sided<br>*Only common tests should be described solely by name; describe more complex techniques in the Methods section.*
☐	☒	A description of all covariates tested
☒	☐	A description of any assumptions or corrections, such as tests of normality and adjustment for multiple comparisons
☐	☒	A full description of the statistical parameters including central tendency (e.g. means) or other basic estimates (e.g. regression coefficient) AND variation (e.g. standard deviation) or associated estimates of uncertainty (e.g. confidence intervals)
☐	☒	For null hypothesis testing, the test statistic (e.g. *F*, *t*, *r*) with confidence intervals, effect sizes, degrees of freedom and *P* value noted<br>*Give P values as exact values whenever suitable.*
☒	☐	For Bayesian analysis, information on the choice of priors and Markov chain Monte Carlo settings
☒	☐	For hierarchical and complex designs, identification of the appropriate level for tests and full reporting of outcomes
☒	☐	Estimates of effect sizes (e.g. Cohen's *d*, Pearson's *r*), indicating how they were calculated

Our web collection on statistics for biologists contains articles on many of the points above.

Software and code

Policy information about availability of computer code

Data collection	No software was used for data collection.
Data analysis	IBD was calculated using hmmIBD v2.0.4<br>Multiple regressions were done using the Python package statsmodels version 0.13.5<br>Networks were visualized using Gephi version 0.9.6 202206221744<br>Custom analysis scripts are available from: https://github.com/glipsnort/Senegal_2019_malaria_analysis

For manuscripts utilizing custom algorithms or software that are central to the research but not yet described in published literature, software must be made available to editors and reviewers. We strongly encourage code deposition in a community repository (e.g. GitHub). See the Nature Portfolio guidelines for submitting code & software for further information.

Data

Policy information about availability of data

All manuscripts must include a data availability statement. This statement should provide the following information, where applicable:

- Accession codes, unique identifiers, or web links for publicly available datasets
- A description of any restrictions on data availability
- For clinical datasets or third party data, please ensure that the statement adheres to our policy

Barcode data are provided as a supplemental file. Sequence data are being uploaded to the NCBI Sequence Read Archive under BioProject PRJNA972644. All genetic

and genomic data from this study are provided by the Senegal Ministry of Health and Social Action and made publicly available, along with the population data used in this study. These population data include the date of sample collection, the name of the health facility where the sample was collected, and the latitude and longitude of the health facility. No other sample information (i.e., related to the individual who provided the sample) was utilized as per the ethical approval for the study.

Research involving human participants, their data, or biological material

Policy information about studies with human participants or human data. See also policy information about sex, gender (identity/presentation), and sexual orientation and race, ethnicity and racism.

Reporting on sex and gender	Data analyzed were from Plasmodium falciparum parasites obtained from individuals who presented to health facilities with fever and who tested positive for malaria infection. No human identifiers were used, including information about sex and gender, as per the study design and ethical approval. The study focuses on parasite populations and uses a subset of infections obtained from any individual who met the inclusion criteria for the study. We do not anticipate any bias among infections from individuals based upon sex and/or gender.
Reporting on race, ethnicity, or other socially relevant groupings	No information about race, ethnicity, or other social grouping was obtained from individuals who presented with malaria infections at health facilities. The study involved analysis of malaria parasite infections from any febrile individual who presented at a health facility who met the inclusion criteria.
Population characteristics	No human population characteristics were used in the study since the study was about malaria infections in the population of individuals presenting to health facility with fever.
Recruitment	Any febrile individual who presented to a health facility that tested positive for malaria infection by rapid diagnostic test or microscopy was ask to consent and/or assent to allow use of a blood sample collected at the time of diagnosis for genetic analysis of malaria parasite material only. Since the samples represented any febrile individual there was no selection bias for sampling. The study was carried out during the peak of malaria transmission, which is seasonal, so samples represent infections from febrile individuals during this collection period. No human genetic material was utilized in the study and all human sequence data obtained during sequencing of the samples was routinely identified as human and removed before making available the malaria parasite genomic data for analysis as per the ethical protocols approved for the study.
Ethics oversight	Ethical approval for the study was first obtained from the Ministry of Health and Social Action in Senegal (Avis Protocol SEN1949) and by the Institutional Review Board (IRB) of the Harvard T.H. Chan School of Public Health (Protocol 16330).

Note that full information on the approval of the study protocol must also be provided in the manuscript.

Field-specific reporting

Please select the one below that is the best fit for your research. If you are not sure, read the appropriate sections before making your selection.

☐ Life sciences ☐ Behavioural & social sciences ☒ Ecological, evolutionary & environmental sciences

For a reference copy of the document with all sections, see nature.com/documents/nr-reporting-summary-flat.pdf

Ecological, evolutionary & environmental sciences study design

All studies must disclose on these points even when the disclosure is negative.

Study description	Study involves the use of blood samples taken at the time of diagnosis of Plasmodium falciparum (malaria) infection at Health Facilities across Senegal to determine the genetic relatedness of the parasites from these infections. Quantitative data was not obtained as part of the study.
Research sample	Blood samples (dried blood from finger stick) obtained during rapid diagnostic testing for Plasmodium falciparum infection were obtained from febrile individuals presenting to health facilities. The rationale was to obtain representative malaria infections from febrile individuals through passive case detection, and dried blood samples are relatively non-invasive, taken at the time of diagnosis, and provide material required for genetic analysis of the malaria parasites from those infections. Dried blood samples were identified by date and health facility only. Nucleic acid material was extracted from the dried blood sample obtained from individuals who assented and/or consented to allow use of the sample for genetic analysis of Plasmodium falciparum detected within the sample. All samples were collected with ethical approval and oversight by the Senegal Ministry of Health and Social Action and Senegal National Malaria Control Program. No datasets were used for the study.
Sampling strategy	The sampling procedure involved obtaining a dried blood sample at the time of diagnosis of Plasmodium falciparum infection as part of routine testing of febrile individuals presenting to health facilities under the Senegal National Malaria Control Program guidelines. Approximately 100 samples were obtained from each health facility, a number of samples that has been previously shown to provide general representation of the malaria parasite population for a health facility. Power calculations for sample size were based upon statistical modeling and simulation of Plasmodium falciparum genetic diversity across a range of transmission levels (determined by incidence) to ensure sufficient power to detect genetic metrics for each study site (health facility).
Data collection	Field teams working with the Senegal National Malaria Control Program collected dried blood spot samples of approximately 100 febrile individuals presenting to each health facility during the malaria transmission season (August to December) after assent/ consent and diagnosis of Plasmodium falciparum infection. Trained nurses and clinic staff obtained the finger stick samples on filter

	paper, recorded the date and health facility location for the sample, and recorded a unique sample identification number (Sample ID). Field staff transported the dried blood samples back to the research laboratory in Dakar, Senegal and registered the sample information related to date of collection and health facility along with the sample identification number (Sample ID).
Timing and spatial scale	Samples were collected during the malaria transmission season from August to December of 2019 at 23 different health clinics from across Senegal that represent different levels of transmission based upon recorded incidence. The latitude and longitude of each health facility is recorded.
Data exclusions	Data were excluded if they did not meet the inclusion criteria in terms of sequencing depth or coverage. Samples were excluded from relatedness analysis if they were classified as polygenomic.
Reproducibility	A small subset of samples (<10%) had genotyping results repeated to evaluate reproducibility. The two data types, molecular barcode genotyping of 24 independent single nucleotide polymorphisms (SNPs) and whole genome sequence data, were generally concordant where both types were available. Details of the concordance are described in the paper.
Randomization	Randomization was not relevant to the study, as we were evaluating a subset of infections per health facility for genetic metrics. All samples were subject to barcode genotyping, and samples chosen for sequencing were selected based on their barcode, so randomization was not applicable.
Blinding	Blinding was not relevant to the study, but analysis was done based upon health facility to compare within and between genetic variation, thus blinding was not carried out.

Did the study involve field work? ☒ Yes ☐ No

Field work, collection and transport

Field conditions	Information including the name of the health facility, incidence, and latitude and longitude is provided in supplementary file. Rainfall changes across Senegal with the rainy season from June to September and ranging from 300 to 1200 mm per year.
Location	Latitude and longitude data are provided in supplementary file to provide the health facility location from which samples were obtained.
Access & import/export	We have permission and ethical approval from the Senegal National Malaria Control Program (Senegal Ministry of Health and Social Action) to use the samples for research of the malaria parasite. We have a CDC Importation Permit that allows us to import the samples for research in the United States.
Disturbance	Samples were collected as part of routine diagnostic procedures carried out at health facilities in Senegal under the guidance of the Senegal Ministry of Health and Social Action and Senegal National Malaria Control Program, thus no disturbance was caused by the study.

Reporting for specific materials, systems and methods

We require information from authors about some types of materials, experimental systems and methods used in many studies. Here, indicate whether each material, system or method listed is relevant to your study. If you are not sure if a list item applies to your research, read the appropriate section before selecting a response.

Materials & experimental systems

n/a	Involved in the study	
☒	☐	Antibodies
☒	☐	Eukaryotic cell lines
☒	☐	Palaeontology and archaeology
☒	☐	Animals and other organisms
☒	☐	Clinical data
☒	☐	Dual use research of concern
☒	☐	Plants

Methods

n/a	Involved in the study	
☒	☐	ChIP-seq
☒	☐	Flow cytometry
☒	☐	MRI-based neuroimaging
